# Deficits in Auditory Rhythm Perception in Children With Auditory Processing Disorder Are Unrelated to Attention

**DOI:** 10.3389/fnins.2019.00953

**Published:** 2019-09-06

**Authors:** Christos Sidiras, Vasiliki Vivian Iliadou, Ioannis Nimatoudis, Manon Grube, Tim Griffiths, Doris-Eva Bamiou

**Affiliations:** ^1^Clinical Psychoacoustics Lab, Third Department of Psychiatry, Neuroscience Sector, Medical School, Aristotle University of Thessaloniki, Thessaloniki, Greece; ^2^Auditory Group, Medical School, Institute of Neuroscience, Newcastle University, Newcastle upon Tyne, United Kingdom; ^3^Faculty of Brain Sciences, UCL Ear Institute, University College London, London, United Kingdom; ^4^Hearing and Deafness Biomedical Research Centre, National Institute for Health Research, London, United Kingdom

**Keywords:** auditory processing disorder, attention, rhythm, auditory perception, isochrony, memory, beat perception

## Abstract

Auditory processing disorder (APD) is defined as a specific deficit in the processing of auditory information along the central auditory nervous system, including bottom-up and top-down neural connectivity. Even though music comprises a big part of audition, testing music perception in APD population has not yet gained wide attention in research. This work tests the hypothesis that deficits in rhythm perception occur in a group of subjects with APD. The primary focus of this study is to measure perception of a simple auditory rhythm, i.e., short isochronous sequences of beats, in APD children and to compare their performance to age-matched normal controls. The secondary question is to study the relationship between cognition and auditory processing of rhythm perception. We tested 39 APD children and 25 control children aged between 6 and 12 years via (a) clinical APD tests, including a monaural speech in noise test, (b) isochrony task, a test measuring the detection of small deviations from perfect isochrony in a isochronous beats sequence, and (c) two cognitive tests (auditory memory and auditory attention). APD children scored worse in isochrony task compared to the age-matched control group. In the APD group, neither measure of cognition (attention nor memory) correlated with performance in isochrony task. Left (but not right) speech in noise performance correlated with performance in isochrony task. In the control group a large correlation (*r* = −0.701, *p* = 0.001) was observed between isochrony task and attention, but not with memory. The results demonstrate a deficit in the perception of regularly timed sequences in APD that is relevant to the perception of speech in noise, a ubiquitous complaint in this condition. Our results suggest (a) the existence of a non-attention related rhythm perception deficit in APD children and (b) differential effects of attention on task performance in normal vs. APD children. The potential beneficial use of music/rhythm training for rehabilitation purposes in APD children would need to be explored.

## Introduction

Auditory processing disorder (APD) is defined as a specific deficit in the processing of auditory information along the central auditory nervous system, including bottom-up and top-down neural connectivity (Iliadou et al., 2017b) and is currently classified in the international statistical classification of diseases and related health problems, 10th edition (ICD-10) as H93.25. APD is linked to functional abnormalities and lesions beyond the cochlea (Musiek et al., 2005b; Gilley et al., 2016; Iliadou and Eleftheriadis, 2017). Children with APD present a wide range of auditory symptoms, and most commonly problems with speech recognition in noise (American Speech-Language-Hearing Association [ASHA], 2005; American Academy of Audiology [AAA], 2010; British Society of Audiology [BSA], 2011). Comorbidity with other neurodevelopmental disorders is often present (Dawes and Bishop, 2009; Serrallach et al., 2016) consistent with the definition of disorders as elaborated in DSM-5 (American Psychiatry Association, 2013).

Even though musical perception comprises a big part of audition, testing music perception in APD populations has not yet gained wide attention in research. Evidence exists that music perception may be compromised in APD. Poor musical ability and/or appreciation of music is a common selfreported symptom by children with APD (American Academy of Audiology [AAA], 2010). Olakunbi et al. (2010) reported poorer meter perception and Scheffner et al. (2017) reported poorer rhythm reproduction in children with APD compared to neurotypical ones. Dyslexic children also show compromised non-musical auditory processing and auditory rhythm perception (Goswami et al., 2013; Colling et al., 2017) while Huss et al. (2011) found these measures to be correlated. Dyslexia may be present in a subgroup (25%) of children with APD (Iliadou et al., 2009).

The focus of this study is on rhythm processing in children with APD. A stimulus is considered rhythmic when periodicities between acoustic events exist. The total percept of a rhythm typically exceeds that of the sum of the processing of each acoustic event, when periodicities are present but not in their absence. For example, a fairly long sequence of identical isochronous beats is likely perceived as a sequence of alternating strong and weak beats, referred to as the “ticktock” phenomenon (Brochard et al., 2003; Abecasis et al., 2005; Potter et al., 2009). Rhythmicity is thought to induce neural oscillations that entrain with the periodicities of the incoming stimuli (Giraud and Poeppel, 2012; Picton, 2013; Andreou et al., 2015) and has been repeatedly demonstrated to produce fluctuations in attention (dynamic attention theory; Jones and Boltz, 1989; Large and Jones, 1999; Escoffier and Tillmann, 2008; Escoffier et al., 2010; Bolger et al., 2013, 2014; Miller et al., 2013; Meltzer et al., 2015; Sidiras et al., 2017).

Perception of rhythm requires the engagement of beat-based timing mechanisms (Teki et al., 2011a, b). Brain areas that have been identified to play particular roles in timing include those traditionally regarded as parts of the central nervous auditory system, as well as motor and cognitive areas. Specifically, beat-based perception involves the striato-thalamo-cortical network, while the superior temporal gyrus in particular responds to omitted beats in isochronous auditory events (Andreou et al., 2015; Lehmann et al., 2016), and the auditory brainstem is sensitive to temporal regularities (Nozaradan et al., 2018). Subcortical as well as cortical structures seem to support auditory rhythm perception.

The primary objective of this study is to measure perception of a simple auditory rhythm in children (sequense of isochronous beats) diagnosed with APD via behavioral psychoacoustic testing and to compare their performance to age-matched normal controls. This type of stimulus invokes the simplest form of rhythm perception (Lerdahl and Jackendoff, 1983; Povel and Essens, 1985) and thus minimizes cognitive and memory load (see also Chermak et al., 2017), and consequently the involvement of non-auditory cognitive related brain areas. The isochrony task (Grube et al., 2010) was used for this purpose in the present study. In this test, the individual ability to detect small deviations in an otherwise isochronous beat sequence is assessed. Isochrony tests have been administered by Grube et al. (2010) in normal young adults, adults with spinocerebellar ataxia (Grube et al., 2010), in children with reading and writing impairments (Grube et al., 2012, 2014), patients with dysfunctions of the basal ganglia (Cope et al., 2014a, b), and patients with Primary Progressive Aphasia (Grube et al., 2016). These studies suggest that the isochrony and related beat-based tasks are (a) highly sensitive to basal ganglia dysfunction (Cope et al., 2014b) but not necessarily to any cerebellar disruption (Grube et al., 2010) in relation to aphasic deficits, whilst also correlating with reading ability in both children and adults (Grube et al., 2013). (b) Correlates with reading ability and phonological skills in children (Grube et al., 2012, 2014).

The primary hypothesis of this study is that performance of children with APD in the isochrony task will be impaired compared to neurotypically developing children. The secondary hypothesis concerns the interaction between cognition and auditory processing (Iliadou et al., 2017b, 2018a; see also Gyldenkærne et al., 2014). There is indication that cognitive factors such as auditory attention, working memory and non-verbal intelligence correlate with performance in psychoacoustic tests (Moore et al., 2010, 2013; Tomlin et al., 2015; Iliadou et al., 2017a, 2018c; Stavrinos et al., 2018). The direction of causality is unclear. Differences between studies may be the result of clinical and control groups used. Moore et al. (2010) measured auditory processing in children and found “that response variability, reflecting attention, and cognitive scores were the best predictors of listening, communication, and speech-in-noise skills” for typically developing school aged children. They then suggested that APD is primarily an attention problem. Tomlin et al. (2015) tested suspected (not diagnosed) APD children and typically developing ones and found significant associations between AP and cognitive scores (sustained attention, auditory working memory, and non-verbal intelligence). Iliadou et al. (2017a) found that “a measure of temporal resolution may offer an early measure reflecting left temporal cortical thinning associated with the transition between Mild Cognitive Impairment and Alzheimer’s disease.” This study stresses the sensory aspect of a specific auditory processing test. Stavrinos et al. (2018) measured different types of attention in APD diagnosed children and found that “the majority of APD children are not at risk of broader attentional problems, but instead face major deficits in auditory attention tasks.” Bamiou et al. (2012) focuses on the functional decline related to auditory processing deficits in stroke patients that might limit their ability for community reintegration. In light of these studies, the second hypothesis in this study is that potential poor performance in rhythm perception of children with APD will not be fully explained by deficits in cognition.

## Materials and Methods

### Participants

Thirty-nine (28 males) children with APD (mean age = 9.3, *SD* = 2,04) and 25 (12 males) control children (mean age = 8.7, *SD* = 1,91), aged between 6 and 12 years participated in this study. ANOVA showed no statistically significant difference in age between groups (*F* = 1.31, *p* = 0.257). None of the children had any previous formal musical education other than the standard school musical curriculum (1 h once a week). Children with APD were referred for listening and academic problems by speech pathologists and/or educators, and were diagnosed with APD in the Psychoacoustic Clinic in AHEPA hospital of Thessaloniki. Diagnosis was made via a standardized clinic psychoacoustic test battery and based on their performance deficit of 2 SDs from the mean of age-matched children on at least two tests on at least one ear (criterion set by American Academy of Audiology [AAA], 2010; Iliadou et al., 2017a; also see Musiek et al., 2005a; Spaulding et al., 2006; Shinn and Musiek, 2007; Turner and Hurley, 2009). Three children with APD were diagnosed with an additional comorbid developmental disorder: attention deficit hyperactivity disorder (ADHD) in 2 cases and developmental language disorder (DLD) in 1 case. All participants presented with normal hearing sensitivity bilaterally as revealed by pure-tone audiometry (PTA) thresholds of 15 dB HL or better at all octave frequencies between 250 and 8000 Hz. Parents and caregivers gave written informed consent for participating in the study, in accordance with the World Medical Association’s Declaration of Helsinki.

### Testing

Testing included SinB test, the isochrony task, and two cognitive tests, a working memory test (Digit Span) and a sustained auditory attention test (SAA). Digit Span was delivered in a sound-treated room via headphones (TDH-50P) at 60 dB HL through a CD player routed via a GSI 61 audiometer. Isochrony task was delivered through a laptop and headphones (Sennheizer, HD PRO 380 pro) and the attention task was delivered through loudspeakers, both at 60 dB HL. 60 dB HL was chosen as an average everyday life intensity presentation of speech stimuli.

#### Speech-in-Babble (SinB) Test

SinB (Iliadou et al., 2009; Sidiras et al., 2016) is a monaural speech in noise test in which participants are instructed to repeat the word they heard after each trial. 50 bi-syllabic words are presented in 7 Signal-to-Noise Ratios (SNR; 7 dB, 5 dB, 3 dB, 1 dB, and −1 dB). Each word is preceded by a short verbal attentional cue (“pite tin lexi,” i.e., “say the word”). The outcome measures are the SNRs in which 50% of the words are correctly identified (SinB_RE and SinB_LE for right and left ear, respectively). Failure in this test is defined scoring above 1.33 dB based on performance of typical development children (Sidiras et al., 2016) and standard audiological criteria (Musiek et al., 2005a; Spaulding et al., 2006; Shinn and Musiek, 2007; Turner and Hurley, 2009; American Academy of Audiology [AAA], 2010; Iliadou et al., 2017b).

#### Isochrony Task

**FIGURE 1:**
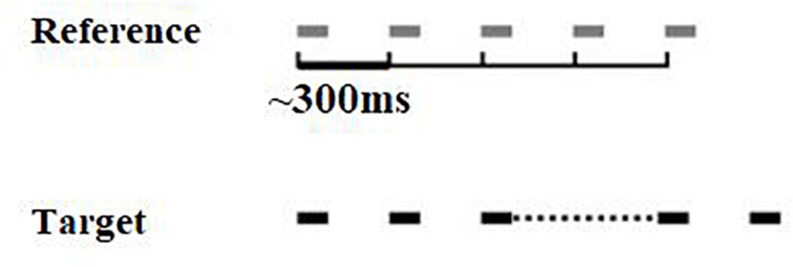
Visual representation of the isochrony task stimuli. Two 5-beat isochronous sequences are presented, in one of which (the target) the interval between the 3rd and 4th beats is lengthened.

The isochrony task is an adaptive (2 alternative forced-choice, 2down-1up) beat perception test developed by Grube et al. (2010). In each trial two 5-beat sequences are presented, one of which is isochronous, while the other one is isochronous except for the lengthened time interval between the third and the forth beat (see Figure 1). The initial (percentage) lengthening was 60% and adaptively changed in steps of 6%, turning to 2% following 4 reversals. Absolute lengthening (in ms) was the product of inter onset-interval (which was not fixed but roved between trials) times the percentage lengthening, e.g., if a certain trial’s inter onset-interval was 400 ms and percentage lengthening 60%, the absolute lengthening was 240 ms. Isochrony score units are percentages that can be translated into ms. Each beat is a pure tone with a frequency of 500 Hz and a duration of 100 ms. The inter onset-interval between the two beats ranges from 300 to 500 ms in 50 ms steps that are randomly roved between the trials. This random variation was used to avoid the formation of learned representations of duration of the reference. It can also “constrain strategies used by the subject to accomplish the task: roving of the tempo between sequences in the metrical rhythm task prevented possible strategies based on focusing on specific intervals as opposed to the overall rhythm” (Grube et al., 2012). The two sequences are presented in random order, with an inter-sequence-interval equal to 1800 ms. A visual alerting cue was offered by the tester for younger children, in order to indicate the presentation of the first and the second sequence (one finger and two fingers). The task is to identify the sequence that contains the lengthening, by orally responding “one” or “two,” according to whether the sequence was presented first or second, respectively. The isochrony task thus measures only the simpler component of rhythm perception, i.e., beat perception (pulse) ability, rather than more complex components, such as grouping structure (for details on components of rhythm see Fitch, 2013). Scores were calculated as the mean of the last six reversals, estimating the 70.7% correct threshold of the psychometric function for the percentage of lengthening detected. This percentage score can be translated to absolute time in ms according to the formula [absolute time = ∼ 400 ^∗^ score]. The isochrony task was delivered through a laptop and headphones (Sennheiser HD 380 pro) at 60 dB sensation level (SL, i.e., relative to the pure tone threshold of the typical listener).

#### Digit Span

Digit Span is a classic test of Working Memory, part of the Wechsler Intelligence Scale for Children (Wechsler et al., 2003). A pre-recorded Greek version (Iliadou et al., 2018b) was implemented in which only one sample of each number was used. That is, the test’s audio file was constructed from a total of 9 recordings which correspond to 9 numbers (1 through 9). This way, possible confounds due to fluctuations in digits’ pronunciation utterance were eliminated. There are two conditions in the Digit Span: forward and backward. In the forward condition, the child listens to and repeats a sequence of numbers. In the backward condition, the child listens to a sequence of numbers and repeats them in reverse order. Each trial is considered successful if all digits are repeated in the correct order. The length of the first sequence of numbers is 2, and progressively increases by one number after each second presentation (i.e., 2, 2, 3, 3, 4, 4, etc digits). The test is terminated when two sequences of the same number of digits are falsely repeated or not repeated at all. The outcome measure is the total number of correctly repeated sequences in forward condition, plus the total number of correctly repeated sequences in backward condition. There are 16 and 14 sequences in the forward and the backward condition, the last one corresponding to 8 and 7 digits, respectively. The best possible score is 30, while in practice primary school children (aged from 6 to 11) do not usually achieve any better than 15.

#### Sustained Auditory Attention Test (SAA)

The Sustained Auditory Attention standardized test consists (Simos et al., 2007) of 180 words, that include 30 random presentations of the target word “milo,” i.e., “apple.” Words are presented by a loudspeaker at ∼60 dB HL, at a rate of one word per second. An image with 4 objects, an apple, a banana, a grape and a watermelon, is presented on a computer screen. The task is to point with a finger to the apple on the screen, each time the target word is presented. Each correct timely response (before the next word is presented) corresponds to 2 points, and 1 point for each late response (during the next 2 words). All other responses i.e., pointing to the wrong object, or responding to a non-target word, are considered false alarms, each corresponding to −1 points. The total score is the points of correct responses minus the number of false alarms. Perfect score, that is, 30 timely responses without any false alarms is 60.

#### Data Acquisition

All children in the APD group completed both the isochrony task and SinB test. All children in the control group completed the isochrony task and most of them the SinB test. Due to time constraints, half children in both groups completed the Digit Span and SAA tests. See Table 1 for details. Mann-Whitney test revealed no significant difference in isochrony scores between children completing vs. those that did not complete Digit Span (*U* = 64.0, *p* = 0.542; *U* = 148.0, *p* = 0.279, for control and APD children, respectively) and SAA (*U* = 61 *p* = 0.403; *U* = 170.5, *p* = 0.588, for control and APD children, respectively).

**TABLE 1:** *N* and percentage of children that completed each tests for APDs and controls.

	**APD (*n* = 39)**		**Controls (*n* = 25)**	
	***N***	**Percentage (%)**	***N***	**Percentage (%)**
Isochrony	39	100	25	100
SinB	39	100	18	72
Digit Span	17	43.6	15	60
SAA	20	51.3	14	56

*SinB, speech in babble; DD, dichotic digit; GIN, gaps-in-noise; RGDT, random gap detection test; PPS, pitch pattern sequence; SAA, sustained auditory attention.*

### Statistical Analysis

Results did not follow a normal distribution under the criterion of skewness and kurtosis with *z* values ranging between −1.96 and 1.96 (Cramer and Howitt, 2004; Doane and Seward, 2011). Non-parametric tests were used for statistical analysis. Mean deviation from median (MD) was used as a non-parametric measure instead of Standard Deviation.

## Results

### Isochrony Performance

**FIGURE 2:**
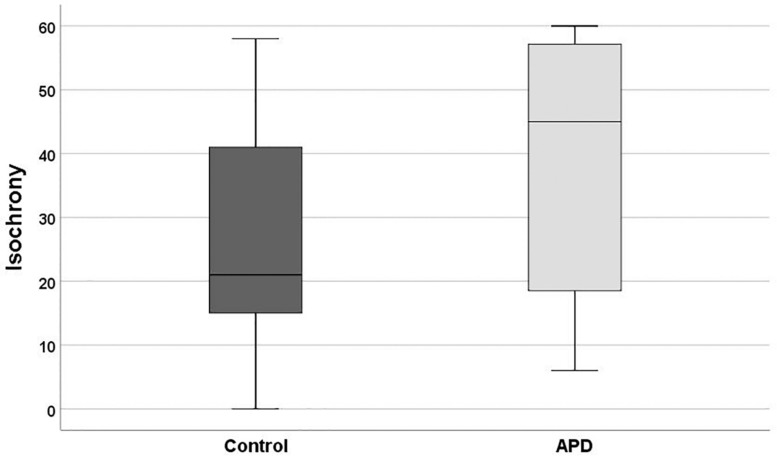
Boxplot of the isochrony score of Controls and APDs. Lower scores indicate better performance. Lower and upper whiskers correspond to minimum and maximum values, the box represents the interquartile range (between 25 and 75%) and the horizontal line represents the median. Units of the isochrony scores are percentages, not ms. e.g., the median isochrony score for the APD group was 45% which translates in (45/100) ^∗^ 400 ∼ = 180 ms. The median isochrony score for the control group was 21% which translates in (21/100) ^∗^ 400 ∼ = 84 ms.

Descriptive statistics and box plots of isochrony performance for APD and control children are shown in Figure 2 and Table 2, respectively. The children with APD performance in isochrony (median = 45) is significantly worse, that is higher scores, compared to controls (median = 21) at *p* = 0.015 (Mann-Whitney test *U* = 311.5). The same analysis was executed, excluding the 3 cases of children with APD and an additional comorbid disorders (i.e., the 2 cases with ADHD and one case with DLD). This did not change the results, as there was a significant difference between the groups (*U* = 274.5, *p* = 0.01).

**TABLE 2:** Isochrony descriptive statistics for APD and control children.

	**Isochrony Scores**	
	**APD (*n* = 39)**	**Controls (*n* = 25)**
Median	45	21
1st quartile	18	14.5
3rd quartile	57.3	41.5
MD	18.49	12.76
Min	6	6
Max	60	58

### Isochrony Performance and Age

**FIGURE 3:**
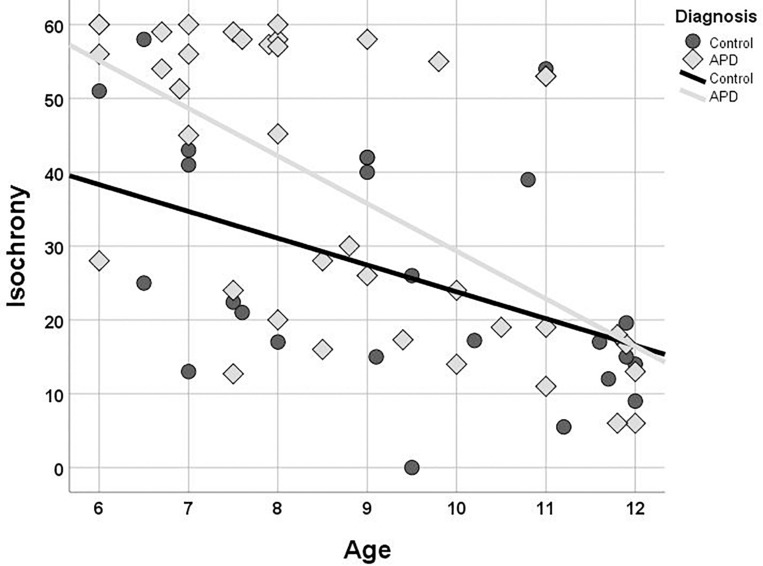
Scatterplot of isochrony scores vs. age for children with APD and controls. In both groups the two measures are correlated (*r* = –0.496, *p* < 0.001 and *r* = –0.409, *p* = 0.005, and for APDs and controls, respectively). Units of the isochrony scores are percentages, not ms. e.g., the median isochrony score for the APD group was 45% which translates in (45/100) ^∗^ 400 ∼ = 180 ms. The median isochrony score for the control group was 21% which translates in (21/100) ^∗^ 400 ∼ = 84 ms.

Isochrony performance improves (i.e., scores decrease) as age increases for both children with APD and controls (see Figure 3). The Kendall’s correlation between performance and age is statistically significant for both groups (*r* = −0.496, *p* < 0.001 and *r* = −0.416, *p* = 0.004 and for APDs and controls, respectively). The two groups differ in performance, however, their difference is not constant across ages, as it is larger in younger ages and progressively decreases to almost no difference at all in older children. This pattern, i.e., difference in performance between control and APD children progressively decreasing as age increases, has been also found for word in noise recognition (see Sidiras et al., 2016) and further analysis was executed to assess for statistical significance. Linear regression and test of parallelism were executed to assess the difference between beta values, i.e., the slopes of the two lines, yielding no significant results (*z* = 0.37, *p* > 0.05, n.s.; Fisher, 1921).

Isochrony scores follow a bimodal distribution and there are some concerns that a ceiling effect may be present for the APD group potentially because in our isochrony task the maximum lengthening of the stimulus interval was capped at 60% (see Figure 3). These properties of our data might compromise the robustness of the correlation statistic result, hence additional statistics were run assessing furtherly for the age effect on isochrony performance. APD scores were divided into two groups around the median (=45). This resulted into two groups, isochrony-low (i.e., better performance; *n* = 19) and isochrony-high (i.e., worse performance; *n* = 20). Mann-Whitney test was executed in order to assess for differences in age between the two groups. Results verified correlation analysis results, as isochrony-low group was 2 years older than isochrony-high group (mean = 9.7, *SD* = 1.81 vs. mean = 7.76, *SD* = 1.47; *U* = 72, *p* < 0.001).

### Isochrony and Cognition

**FIGURE 4:**
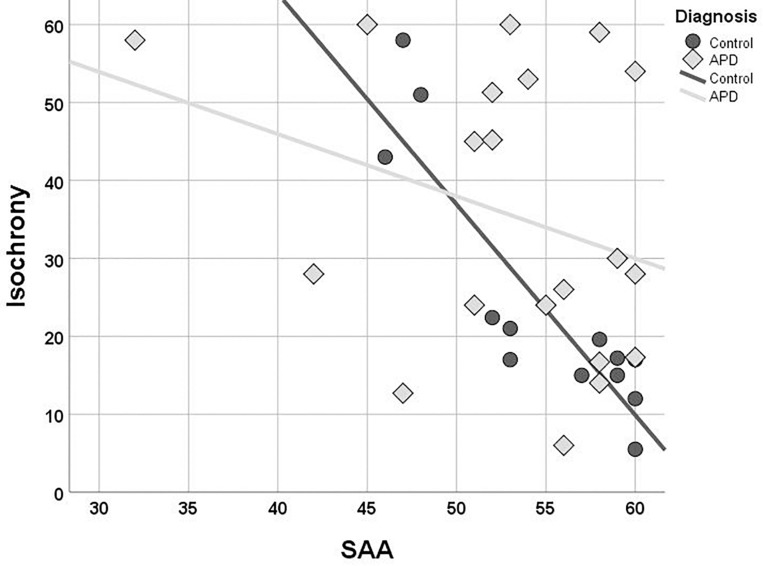
Scatterplot of isochrony vs. SAA for children with APD and controls. Correlation between the two measures is significant for controls, but not for APDs (*r* = –0.701, *p* = 0.001 and *r* = –0.130, *p* = 0.433, n.s., respectively). SAA, sustained auditory attention. Units of the isochrony scores are percentages, not ms. e.g., the median isochrony score for the APD group was 45% which translates in (45/100) ^∗^ 400 ∼ = 180 ms. The median isochrony score for the control group was 21% which translates in (21/100) ^∗^ 400 ∼ = 84 ms.

Children with APD scored slightly worse than controls in both working memory (Digit Span; median = 9 and median = 10, respectively) and sustained attention (SAA; median = 54.5 and median = 57.5, respectively) but these differences were not statistically significant (*U* = 115, *p* = 0.396 n.s., for Digit Span and *U* = 87,5, *p* = 0.126 n.s., for SAA). The two groups differed in terms of correlation between isochrony and attention. While correlation of attention and isochrony was strong in controls (Kendall’s *r* = −0.701, *p* = 0.001), which corresponds to nearly half of the variance in the performance, the effect was absent in children with APD (*r* = −0.130, *p* = 0.433, n.s.). As shown in Figure 4, there are several children with APD that scored high (i.e., showed good performance) in the attention test, but still failed in isochrony. Working memory on the other hand did not correlate significantly with isochrony either when the two groups were examined merged (*r* = −0.225, *p* = 0.087) or separately (*r* = −0.176, *p* = 0.352 APDs and *r* = −0.270, *p* = 0.175 controls).

Due to considerations about the robustness of correlation test results within the APD children group due to the properties of their isochrony scores’ distribution (i.e., bimodal distribution and concerns about ceiling effect; see above “isochrony performance and age”), additional statistics were executed in order to assess furtherly for effects between cognition and isochrony performance. Mann-Withney test verified correlation analysis results for APD children, as revealed no differences between the isochrony-High and isochrony-Low groups in both working memory (*U* = 25, *p* = 0.319) and sustained attention (*U* = 32.5, *p* = 0.195).

### Isochrony and Speech in Noise Recognition

**FIGURE 5:**
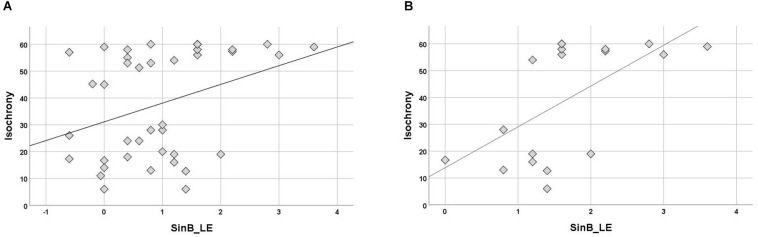
**(A)** Scatterplot of isochrony vs. SinB_LE for APDs. Correlation between the two measures is significant (*r* = 0.245, *p* = 0.034). SinB_LE, SinB left ear. **(B)** Scatterplot of isochrony vs. SinB_LE for APDs who failed in SinB (right and/or left ear). Correlation between the two measures is significant (*r* = 0.445, *p* = 0.014). SinB_LE, SinB left ear. Units of the isochrony scores are percentages, not ms. e.g., the median isochrony score for the APD group was 45% which translates in (45/100) ^∗^ 400 ∼ = 180 ms. The median isochrony score for the control group was 21% which translates in (21/100) ^∗^ 400 ∼ = 84 ms.

Isochrony correlation with SinB (right, left ear, and mean of two ears) was computed for separate groups. No correlation was significant in controls. In the APD group, isochrony correlated only with SinB_LE (Kendall’s *r* = 0.245, *p* = 0.034; see Figure 5A). Further analysis were executed for the APD group for children that failed in SinB (either SinB_RE, SinB_LE or both; *n* = 18). Failure in SinB is defined as scoring above (worse) 1.33 dB in at least one ear. This criterion is based on performance of typically developing children (see Sidiras et al., 2016) and standard audiological criteria (2 SD worse performance from mean; Musiek et al., 2005a; Spaulding et al., 2006; Shinn and Musiek, 2007; Turner and Hurley, 2009; American Academy of Audiology [AAA], 2010; Iliadou et al., 2017b). Isochrony correlated with SinB_LE (*r* = 0.445, *p* = 0.014; see Figure 5B).

Due to considerations about the robustness of correlation test results within the APD children group due to the properties of their isochrony scores’ distribution (i.e., bimodal distribution and concerns about ceiling effect; see above “isochrony performance and age”), additional statistics were executed in order to assess furtherly for effects between SinB_LE and isochrony performance. Two Mann-Withney tests were executed, respectively to the correlation statistic tests, assessing for differences in SinB_LE scores between isochrony-High and isochrony-Low group. In the first analysis, all APD children were fed into the analysis, in the second one, only the APD childred that failed in SinB (SinB > 1.33 dB in at least one ear). The first analysis did not yield significant results between the two isochrony groups (*U* = 136.5, *p* = 0.131), but the second one revealed differences verifying the correlation analysis: isochrony-Low (better performance) group scored lower (better) scores in SinB_LE, compared to the isochrony-High (worse performance) group (median = 1.2 dB vs. median = 1.9 dB, respectively; *U* = 8, *p* = 0.004).

## Discussion

In this study, we tested via psychoacoustic testing the hypothesis that children with APD present with rhythm perception deficits, since children with APD manifest compromised music skills). This was done by comparing their performance to the performance of children without APD in a test with the simplest rhythm possible (sequence of isochronous beats). This stimulus was selected in order to minimize any attention and memory confounds. Our hypothesis was confirmed since children with APD scored worse compared to the age-matched control group. Comorbidity of APD with other neurodevelopmental disorders (Dawes and Bishop, 2009; Serrallach et al., 2016) may affect rhythm performance, however, excluding children with comorbidities in our sample (*N* = 3) did not affect the result. Due to the presence of a only small number of these children in the present study, the discussion is focused on the APD population, particularly as there are relatively fewer studies in the field. Our aim here is to specifically discuss results comparing controls and children diagnosed with APD.

### Isochrony and Age

Performance improved as age increased for both APD and control children. This was an expected result, as neuromaturation and learning occurs over time and it is in accordance with previous research (Paananen, 2006). However, this improvement was larger for children with APD, even though not enough to be statistically significant. This difference has been found also present in SinB performance (Sidiras et al., 2016) and suggests a different pace of maturation rhythm perception in APD compared to controls for this age. In particular, children with APD perform way worse than controls in smaller ages (i.e., 6–7 years old), but this difference gets smaller in the end of primary school years (i.e., 12 years old). Thus, children with APD, present with a delay in maturation until the first primary school years, which is compensated (partially or maybe completely in some cases) by rapid maturation pace (compared to children with typical development) in later ages. However, it is crucial to point out that the period during which auditory processing is compromised may have detrimental and possibly irreversible effects in the knowledge and academic/social skill acquisition, and psychological development for the individual child (Bamiou et al., 2001; Iliadou et al., 2009; Valente et al., 2012; Lucker, 2015). A possible limitation of the study regarding this interpretation is the bimodal distribution of isochrony scores which might compromise the robustness of the correlation analysis. Additional analysis verified these results thus favoring our original interpretation on neuromaturation.

### Isochrony and Cognition

Assessing the relationship between cognition and rhythm perception was the second purpose of this study. Two measures of cognition were implemented, working memory (Digit Span) and sustained auditory attention (SAA). Both tests supported our hypothesis, that is, poor performance in isochrony in children with APD is not merely a result of poor cognition.

The Digit Span did not predict performance in isochrony in either group (i.e., APDs and controls) either in merged or in separate group analysis. This result may seem counter-intuitive at first sight, given the comparison of the two sequences in the isochrony task that would require storage and processing in working memory of a total of 10 auditory events with a total mean duration of 5.2 ms, or alternatively 8 inter-beat durations. Our negative result suggests that Isochrony has a low load on working memory resources. This might be due to neural entrainment induced by the isochrony stimuli, which may interfere with stimuli’s encoding. It may be hypothesized that neural synchronization to the rhythmic auditory stimuli and efficient auditory processing of rhythm leads to “easier” sensory perception less depended on cognition. Another possibility is the interaction between neural oscillations and working memory, which have been shown to be correlated with working memory (Wilsch and Obleser, 2016). Auditory rhythm produces slow neural oscillations in the auditory cortex that are similar to the rhythm itself in frequency (Henry and Obleser, 2012; Henry et al., 2014; Andreou et al., 2015; Herrmann et al., 2016; Bauer et al., 2018). Therefore, it is plausible that neural oscillations produced by isochrony may enhance the capability of working memory to process such a long in duration auditory signal. However, this is not supported by the results of the current study as there would be a correlation between ability to perceive rhythm and working memory.

Sustained auditory attention predicted performance with a great amount of variance (nearly half) of isochrony performance in control children, a result that highlights the interplay of attention, and auditory processing as others have reported in typically developing primary school children (Moore et al., 2010). In APD children however, this effect was absent with *r* = −0.130 (see also Stavrinos et al., 2018). This result strongly suggests the presence of factors other than sustained auditory attention being responsible for the isochrony performance in children with APD.

It is important to stress that a uniform effect of attention on auditory processing tests across children with vs. children without APD should not be taken as a given, and should always be checked. This result questions studies concluding that “APD is primarily an attention problem” (Moore et al., 2010). Findings of attention and other cognitive factors as being the best predictors of auditory processing performance of general population school children should not be extrapolated to children diagnosed with APD.

### Isochrony and Speech in Noise Recognition

Results in this study showed a correlation of isochrony with speech in babble perception which was statistically significant for the left ear performance in the APD diagnosed group. The correlation was larger for children failing the SinB test. This may be interpreted as an association of the ability to perceive words in babble with the ability to perceive rhythm. This association was absent in the control group and its presence in the APD group was limited to the left ears performance. Asymmetry between right and left ears is not uncommon and it is found in different audiometric tests (McFadden, 1993; Khalfa and Collet, 1996; Khalfa et al., 1997; Veuillet et al., 2007; DeLeon et al., 2012). Correlation of OAE suppression and SinB failure in APD diagnosed children has been recently documented (Iliadou et al., 2018d) indicating a possible lesion at the level of the brainstem and beyond.

The specific correlation of isochrony with left (but not right) SinB may additionally be linked to the differential tuning of the right vs. left auditory cortex. The right auditory cortex (where the left ear mainly projects) is tuned at 4 Hz, while the left auditory cortex is tuned at 25 Hz (Poeppel, 2003; Boemio et al., 2005; Giraud et al., 2007). The right auditory cortex preferential tuning is explained by studies on speech acoustics as a focus on the temporal characteristics of speech and specifically on the syllabic rate (Edwards and Chang, 2013; Picton, 2013; Overath et al., 2015). In this sense, isochrony and SinB may be tapping into similar temporal processes taking place at the right auditory cortex. Interestingly their correlation is enhanced when focusing on those children diagnosed with APD that have documented speech in babble deficits. As a future endeavor, it might be useful to investigate the effects of rhythmic training on SinB and the rehabilitation potential of such training.

### Why APD Children Perform Poor in Rhythm Perception – A Neurobiological Approach

Rhythm perception, in particular perception of isochronous sequences of beats involves several mechanisms and brain areas. Neural oscillations are invoked at the level of the auditory cortex (Giraud and Poeppel, 2012; Picton, 2013; Andreou et al., 2015) when such a stimulus is perceived.

In terms of the predictive coding theory (Kumar et al., 2011), a prediction about the incoming signal is produced as a bottom-up process which is then passed on to lower levels of the sensory processing hierarchy by backward connections to be compared with the low-order representations (i.e., the sensory input at the lowest level). This comparison yields a prediction error which is passed by forward connections to higher levels. This process if further supported by neural oscillations as dynamic priors, when rhythm is detected, (Friston and Kiebel, 2009), and as the correlation between isochrony and attention task performance in the normal group would suggest. Sensory trajectories convey the ability to recognize sounds in a sequence leading to good levels of prediction errors.

In order for a prediction error to be generated in the case of rhythm perception, both neural oscillations and the representation of the isochronous/delayed incoming beat sequence should be intact. Neural synchrony, that is, neural impulses generated by the stimuli arriving at the same time, at the level where prediction error is supposed to be generated, is dependent on phase locking within the central auditory pathway and correlates to temporal processing (Harris and Dubno, 2017). Thus, deficits in this temporal processing mechanism could be a sufficient cause for degraded representation of the incoming beat sequence, and consequently absence of generation of prediction error for the non-isochronous beat sequence, in children with APD. However, since both subcortical and cortical auditory areas are sensitive in temporal regularities of auditory stimuli (Andreou et al., 2015; Lehmann et al., 2016; Nozaradan et al., 2018), impairments in these areas in children with APD might lead to absence of generation of prediction error for the non-isochronous beat sequence and then to degraded neural oscillations or even absence of them, resulting also to the poor performance in the isochrony task. We thus propose that attentional recruitment does not take place in the APD group due to significant prediction errors leading to perceived violations from isochrony. This would be supported by our findings that attention and working memory, which takes place in higher levels, were not significantly different in the APD vs. the normal control group, and did not correlate with task performance in the APD group. We would thus argue that the isochrony task poor performance lies in a prediction error bottom-up process influencing top-down (empirical prior) leading to failure to perceive the rhythmic difference.

### Clinical Use of Isochrony

Isochrony was found to be a test that can distinguish between children with APD and the normal population in this study. This indicates that it is a promising clinical test, with a potential high diagnostic value once age adjusted norms are established. The normative process would have to include a larger number of children for each age group than the present study. It should also be noted that parameters of the isochrony task might play a role regarding performance results. The choice of IOIs of 300–500 ms by the team who developed the test used in our study (Grube et al., 2010) was based on the fact that these correspond to time intervals between stress events in speech and musical beats (Scott, 1982; Rosen, 1992; Drake et al., 2000), and that the auditory cortex is “tuned” to frequencies around 4 Hz that reflect the syllabic rate (Edwards and Chang, 2013; Picton, 2013).

Our study scope was not to investigate what would be the best paradigm to investigate this temporal function, but to apply a pre-existing test on a clinical population that would be expected to show deficits. Current results, however, indicate that the initial step may be a task parameter that should be modified in the future. The poor performance in a large number of the APD children suggests the presence of a ceiling effect, which does not appear to be present in controls. Future studies should consider changing the initial step of the isochrony task to better characterize isochrony performance in children with APD.

Comorbidity issues should also be further considered. There was a relatively low occurrence (in three out of thirty-nine children) of neurodevelopmental comorbidities in our children with APD, who were recruited from a Psychoacoustic Clinic evaluating for APD. In light of previous published studies on the isochrony task we would expect children with additional comorbidities such as dyslexia (Huss et al., 2011; Goswami et al., 2013; Colling et al., 2017) and ADHD (Puyjarinet et al., 2017) to perform worse on this task than controls. Our results may thus under-represent the true prevalence of these deficits in a “typical” clinical APD population that would have a higher rate of comorbid neurodevelopmental disorders (Dawes and Bishop, 2009). Finally, the correlation of isochrony with attention in control children should be taken under consideration, as abnormal results in the absence of an APD may also be due to primary attention deficits.

### Rhythm Effects on APD – Further Research

Abnormal rhythm perception in children with APD may interfere with rhythm induced effects, as the “ticktock” phenomenon (Brochard et al., 2003; Abecasis et al., 2005; Potter et al., 2009), neural entrainment, and oscillations in attention as described by dynamic attention theory (Jones and Boltz, 1989; Large and Jones, 1999; Escoffier and Tillmann, 2008; Escoffier et al., 2010; Bolger et al., 2013, 2014; Miller et al., 2013; Meltzer et al., 2015; Sidiras et al., 2017) and speech segregation (Giraud and Poeppel, 2012). It is crucial for further research on APD to investigate the extent of compromised processing, as well as other skills that have been shown to correlate with rhythm perception, such as phonological awareness and literacy (Huss et al., 2011; Grube et al., 2012, 2014; Goswami et al., 2013; Colling et al., 2017), as acquisition of all these skills are part of the normal neurodevelopmental course in children.

## Conclusion

In the present study deficits in rhythm perception, compared to normal children were found in children with APD. The performance of the normal control group correlated with auditory attention, while in the APD group this association was missing. These results indicate that poor rhythm perception in APDs is a result of the presence of a genuine sensory processing deficit and not an attention related deficit. It would be worthwhile to investigate further to understand the perceptual/cognitive/sensory interaction, to better inform management and training decision making. Our findings underline the link between rhythm and speech perception, and suggest the potential beneficial use of music/rhythm training for rehabilitation purposes in children with APD.

## Data Availability

The datasets generated for this study are available on request to the corresponding author.

## Ethics Statement

This study was carried out in accordance with the recommendations of the “Ethics and Bioethics Committee of the Aristotle University of Thessaloniki” with written informed consent from all parents and caregivers of all subjects. All parents and caregivers of the subjects gave written informed consent in accordance with the Declaration of Helsinki. The protocol was approved by the “Ethics and Bioethics Committee of the Aristotle University of Thessaloniki.”

## Author Contributions

CS, VI, D-EB, and IN conceived and designed the study, and drafted the manuscript. CS performed the experiment and statistical analysis, and wrote the first draft of the manuscript. VI and D-EB critically revised the drafted manuscript. CS, VI, IN, MG, GT, and D-EB made substantial contribution to the data interpretation, critically revising the drafted manuscript, final approval of the version to be published, and agreement to be accountable for all aspects of the work in ensuring that questions related to the accuracy or integrity of any part of the work are appropriately investigated and resolved.

## Conflict of Interest Statement

The authors declare that the research was conducted in the absence of any commercial or financial relationships that could be construed as a potential conflict of interest.
